# Agraphia in Bulbar-Onset Amyotrophic Lateral Sclerosis: Not Merely a Consequence of Dementia or Aphasia

**DOI:** 10.3233/BEN-2008-0219

**Published:** 2009-07-29

**Authors:** Hiroo Ichikawa, Nobuyoshi Takahashi, Soutaro Hieda, Hideki Ohno, Mitsuru Kawamura

**Affiliations:** Department of NeurologyShowa University School of MedicineShinagawa-kuTokyoJapan

**Keywords:** Amyotrophic lateral sclerosis, writing error, agraphia, syntactic error, frontotemporal dementia

## Abstract

The clinical significance and characteristics of writing errors in bulbar-onset amyotrophic lateral sclerosis (ALS) are not clear. We retrospectively investigated writing samples in 19 patients with bulbar-onset ALS without preceding extra-motor symptoms. Co-development of dementia and/or aphasia was also explored and single photon emission computed tomography (SPECT) images of the brain were reviewed. As a result, a high prevalence of writing errors (15 of the 19 patients) was found. Of note were isolated writing errors with neither dementia nor aphasia verified in 2 patients whose dysarthria was mild enough to evaluate spoken language. The remaining 13 patients also showed agraphia, but either dysarthria was too severe to evaluate aphasia or frontotemporal dementia (FTD)-like features co-existed. Of these patients, one who initially lacked dementia subsequently developed FTD-like features. The frequent writing errors were omission or substitution of kana letters and syntactic errors. SPECT images showed bilateral or left-side dominant hypoperfusion in the frontotemporal lobes as a consistent feature. These results show that patients with bulbar-onset ALS frequently exhibit agraphic writing errors and that these are not merely consequences of dementia or aphasia. However, these writing errors may indicate the involvement of frontotemporal language-related areas beyond the primary motor cortex.

## 1. Introduction

Motor neuron disease (MND) and/or amyotrophic lateral sclerosis (ALS) has been documented to accompany varying degrees of cognitive impairment more often than previously considered [1,2,23,26,31,33,40]. Of the cognitive changes in MND and/or ALS (MND/ALS), the most consistent feature has been reported to be executive dysfunction represented by impaired verbal fluency, based on detailed neuropsychological assessments [1,2,23,26,31,33,40]. Besides, impaired confrontation naming has been repeatedly reported with speculation of underlying language dysfunction in MND/ALS [1,2,40], although the affected processes are not completely clear. However, detailed neuropsychological assessments can only be applied for less severely affected patients with MND/ALS. Meanwhile, full-blown dementia associated with MND/ALS, in other word ALS/MND with dementia (ALS/MND-D), is now understood to show typically the feature of frontotemporal dementia (FTD) [28,36], and aphasic syndrome associated with MND/ALS has also been reported as rare cases [4–7,10,17,22,29,38]. In general, dementia is usually recognized to precede motor symptoms, but the opposite observation has also been documented [18, 31]. It is of note that full-blown dementia or aphasic syndrome has been reported most frequently in combination with bulbar-onset MND/ALS [4–7,10,17,22, 23,29,31,33,38], suggesting a strong linkage between such extra-motor and bulbar symptoms.

Bulbar-onset ALS presents with dysarthria which causes early deterioration of spoken language leading

ISSN 0953-4180/08/$17.00 © 2008 – IOS Press and the authors. All rights reserved to mutism and make subsequent evaluation of language functions difficult. However, in contrast to limb-onset ALS, the motor functions of the hand are relatively well preserved in bulbar-onset ALS, which enables evaluation of writing as one aspect of language. Nonetheless, the characteristics of writing problems in bulbar-onset ALS have not been analyzed in detail, and we consider that investigation of writing errors may provide further insights into language functions in patients with bulbar-onset ALS. Recently, we reported a high prevalence of writing errors, suggesting involvement of language dysfunction, in patients with ALS/MND-D, most of whom showed the features of bulbar-onset ALS [18]. This observation raised a further question whether writing errors were resultant or partial features of dementia and/or aphasia (dementia/aphasia), or could appear independently of dementia/aphasia in patients with bulbar-onset ALS. In the present study, we investigated the prevalence, onset time, and characteristics of writing errors in patients with bulbar-onset ALS without preceding extra-motor symptoms including dementia/aphasia. We also studied co-development of dementia/aphasia as motor symptoms progress, to clarify the association between writing errors and these conditions. Additionally, we reviewed single photon emission computed tomography (SPECT) images of the brain to assess the potential lesions and pathomechanisms that may underlie writing errors.

## 2. Methods

### *2.1. Patient selection*

We reviewed the medical records of Japanese patients with MND/ALS who were admitted to our department from May, 1990 to November, 2007, and found 87 patients who fulfilled the clinical and electrophysiological criteria for clinically definite ALS according to the Revised Criteria for the Diagnosis of Amyotrophic Lateral Sclerosis of the World Federation of Neurology Research Group on Motor Neuron Disease: El Escorial revised [41]. Of these 87 ALS patients, bulbar-onset ALS was diagnosed in 27 patients, two of whom were excluded since they had preceding dementia. In the remaining 25 patients, writing samples were searched from medical records, interview forms, diaries, and letters to doctors, as well as from confrontation writing tests. Finally, we employed 19 patients from whom writing samples were obtained. At the time when these samples were written, the patients had the ability to write legible letters with their own hand and could ambulate free from artificial respiratory ventilation

### *2.2. Evaluation of writing errors*

The presence of critical writing errors and the onset-time and characteristics of the writing errors were evaluated in the samples (spontaneous writing and dictation of words and sentences) obtained from 19 patients. To describe these results, we explain the Japanese writing system, which consists of two types of letters: kana (phonogram) and kanji (morphogram). A kana letter represents a spoken syllable, with a strict one-to-one correspondence of sound-to-script. Kana words consist of one or more kana and always have only one phonetic pronunciation; therefore, kana words correspond to regular words in European languages. In contrast, kanji is not used in spoken language. Each kanji letter conveys a particular meaning, but has several different pronunciations. Kanji words consist of more than two kanji letters and usually have one pronunciation attached to the meaning; therefore, Kanji words are comparable with irregular words in European languages. There are 2,000 kanji letters in common use, and these are learned during compulsory education in Japan from age 6 to 15 years old. Today, normal Japanese adults are able to write, read and comprehend these basic kanji letters as well as all kana letters. Generally, a Japanese sentence is written by mixing kana and kanji letters: kanji is usually used for writing most nouns and roots of verbs, adverbs and adjectives, and kana letters are mainly used to write inflectional endings, conjunctions and postpositions (prepositions) that integrate syntax.

The 25 patients in the study had education ranging from 9 to 16 years, and in the premorbid state the patients had ability to write, read and comprehend Japanese letters as well as normal adults. This information about writing ability was confirmed through interviews with their family. The presence of writing errors was defined as three or more repeated mistakes in basic kana or kanji letters, which can be written correctly by students at the end of their first six years of education. To assess the types of mistakes, we evaluated writing errors for both kana and kanji letters in words based on the classification of Sakurai et al. [31]: non-response, partial response, and morphologic, phonologic and semantic paragraphia. Syntactic problems involving mistakes with suffixes or postpositions were also investigated.

### *2.3. Evaluation of dementia and aphasia*

Co-development of dementia and aphasia was evaluated in patients for whom writing errors were found to assess the association of these conditions with the writing errors and to investigate the pathomechanism. Since all the subjects had bulbar-onset ALS, all suffered from dysarthria as an initial symptom followed by dysphagia and atrophy with fasciculation of the tongue. These clinical characteristics sometimes made it difficult to evaluate dementia and aphasia, although the severity differed among the patients. Dementia was diagnosed based on daily activities, and particularly behavioral changes, with reference to the clinical diagnostic criteria for FTD [28,36], and the International Classification of Diseases-10 [42]. To avoid overlooking the possible development of FTD, we focused on behavioral changes including reduced volition, disinhibition, attention deficits, and restlessness. We also performed several neuropsychological tests, including the Mini-Mental State Examination (MMSE), the Hasegawa Dementia Scale-Revised (HDS-R) test, and the Adult Intelligent Scale-Revised (WAIS-R) test for measurement of general cognitive function. The HDS-R is a simple test that is similar to the MMSE and is thought to correlate well with the MMSE [16]. The HDS-R is commonly used to diagnose dementia in Japan, with a score under 20 points (the highest possible score is 20 points) usually considered to indicate dementia. We also used the Frontal Assessment Battery (FAB) as a measurement of frontal lobe dysfunction. Since these neuropsychological tests were not conducted systematically and the timing of the tests was diverse across patients, all examinations were not necessarily performed for all patients. In addition, the tests were executed through writing for some patients whose spoken language was incomprehensible due to severe dysarthria. Aphasia was evaluated if dysarthria was mild enough to allow evaluation of spoken language through spontaneous speech, naming, repetition and auditory comprehension. However, for most patients evaluation of speech was difficult due to the reasons discussed above.

### 2.4. *Evaluation of SPECT images*

SPECT images were acquired with $^{99m}$Tc-ethylcystinate dimmer (ECD), $^{99m}$Tc- hexamethyl propyleneamine oxide (HMPAO), or N-isopropyl-p-[$^{123}$I] iodoamphetamine (IMP). The images were assessed visually to evaluate the lesions that were potentially associated with the writing errors.

## 3. Result

### 3.1. *Prevalence of writing errors and its relationship with dementia or aphasia*

Writing errors were observed in 15 of 19 patients. We were unable to exclude the possibility that the other 4 patients had writing errors because the number of writing samples was relatively small for these patients. The following investigations were performed only in the 15 patients with confirmed writing errors. A summary of the clinical backgrounds of these patients is shown in Table 1. Of theses patients, 2 were autopsied and the diagnoses of ALS were confirmed neuropathologically (a precise investigation of extra-motor brain pathology is now in progress). The onset age of ALS with bulbar symptoms ranged from 43 to 80 years old (mean: 69.3 $\pm$ 8.8 years old). All patients were right-handed. The interval from onset of ALS to the time when the writing errors were first encountered varied from 3 to 40 months (mean: 18.6 $\pm$ 13.3 months). In the 4 patients without confirmed writing errors, the interval from onset of ALS to the time when the samples were written was considerably shorter at 3 to 10 months (mean: 7.3 $\pm$ 3.1 months).

At the time when the writing errors were encountered, dysarthria was too severe to evaluate speech or the speech was completely aphonic in 10 patients (patients 3–6, 8–12 and 15), thereby rendering a detailed evaluation of spoken language impossible. Prior to the time when the samples were written, aphasia had not been noted by family members, doctors, or other medical staff. In the other 5 patients (patients 1, 2, 7, 13 and 14) speech was mildly dysarthric, but sufficiently comprehensible to allow confirmation of absence of aphasia at the time of evaluation.

Only 2 patients had been diagnosed with dementia by testing with the MMSE or HDS-R at the time when the samples were written. However, 9 patients (patients 7–15) showed varying degrees of behavioral changes that suggested development of FTD. The other 6 patients (patients 1–6) showed neither behavioral changes nor decline in neuropsychological tests, indicating the absence of dementia. Of these patients, one patient (patient 6) subsequently developed behavioral changes, and therefore FTD-like behavioral changes have been observed in 10 of 15 patients during the clinical courses to date. In the other 5 patients, the observation period may have been too short to evaluate dementia or the development of dementia is unclear.

Table 1
Summary of background of 15 patients with writing errors

| Patient | Age/Gender | Handedness | Duration from the onset of ALS to writing errors (months) | Aphasia | FTD-like Behavioral change | Neuropsychological tests |
|---|---|---|---|---|---|---|
| 1 | 80 F | Right | 15 | – | – | MMSE 27; FAB 16 |
| 2 | 68 M | Right | 9 | – | – | MMSE 30; FAB 16; WAIS-R 92 |
| 3 | 71 M | Right | 36 | N.A. | – | HDS-R 27 |
| 4 | 68 M | Right | 23 | N.A. | – | HDS-R 27 |
| 5 | 64 M | Right | 6 | N.A. | – | HDS-R 30 |
| 6 | 67 M | Right | 9 | N.A. | –[1] | HDS-R 29 |
| 7 | 71 M | Right | 4 | – | + | MMSE 23; FAB 12 |
| 8* | 73 F | Right | 16 | N.A. | + | HDS-R 26 |
| 9* | 43 F | Right | 40 | N.A. | + | HDS-R 19 |
| 10 | 65 M | Right | 37 | N.A. | + | FAB 15 |
| 11 | 71 F | Right | 23 | N.A. | + | N.A.[2] |
| 12 | 80 M | Right | 18 | N.A. | + | N.A.[2] |
| 13 | 74 F | Right | 4 | – | + | HDS-R 24 |
| 14 | 75 F | Right | 3 | – | + | MMSE 12; FAB 12 |
| 15 | 69 F | Right | 36 | N.A. | + | HDS-R 26; FAB 11 |
| Mean age, 69.3 ± 8.7 | | | 18.6 ± 13.3 | Absent in 6 | Absent in 6<br>Absent in 6 | |

Abbreviations: M, male; F, female; ALS, amyotrophic lateral sclerosis; FTD, frontotemporal dementia; MMSE, Mini-Mental State Examination; Fab, Frontal assessment Battery; WAIS-R, Wechsler Adult Intelligence Scale-Revised; HDS-R, Hasegawa's Dementia Scale-Revised; N.A. not available. Mean ± SD is shown for mean age, and duration at the bottom. N.A. in the aphasia indicates that spoken language was too severely dysarthric to evaluate or completely aphonic at the time when writing errors were encountered (patients 3–6, 8–12, and 15). N.A. in neuropsychological tests indicates that tests were tried but unable to be accomplished.* Autopsied cases. [1] Behavioral change developed 6 months after.

Table 2
Characteristics of writing errors in 15 patients

| Types of writing errors | Patients | | | | | | | | | | | | | | | Total number |
|---|---|---|---|---|---|---|---|---|---|---|---|---|---|---|---|---|
| | 1 | 2 | 3 | 4 | 5 | 6 | 7 | 8 | 9 | 10 | 11 | 12 | 13 | 14 | 15 | of patients |
| Kana | | | | | | | | | | | | | | | | |
| Nonresponse | | | | | | | | | | | | | | | | 0 |
| Partial response | + | + | + | | | + | + | + | | + | + | + | | + | + | 11 |
| Phonologic error | + | + | | + | + | | + | + | | | + | | | | + | 8 |
| Morphologic error | + | | | | | | + | | | + | | | | | + | 4 |
| Semantic error | | | | | | | | | | | | | | | | 0 |
| Syntactic error | | | | | | + | | + | | + | + | | | + | + | 6 |
| Kanji | | | | | | | | | | | | | | | | |
| Nonresponse | | | | | | | | | | | | | | | | 0 |
| Partial response | | | | + | | | | | + | | | | | + | | 3 |
| Phonologic error | + | | | | | | | | + | | | + | | | | 3 |
| Morphologic error | + | | | | | | | | + | | | + | | | | 3 |
| Semantic error | | | | | | | | | | | | | | | | 0 |

It is of note that the two patients (patients 1 and 2) whose dysarthria was mild enough to allow evaluation of spoken language showed isolated writing errors without dementia/aphasia. The errors observed in writing did not appear in spoken language and their reading was perfect. In addition, the patients were very polite, well oriented and well aware of their illness, with excellent scores in neuropsychological tests and a Digit Span Forward score of 7, suggesting lack of dementia and attention deficit. These characteristics suggest a feature of "pure agraphia". Subsequent development of aphasia and dementia remains unclear over the short period after encountering the writing errors.

### 3.2. Characteristics of writing errors

Representative samples of different types of writing errors are shown in Fig. 1 and the characteristics of writing errors observed in 15 patients are summarized in Table 2. As shown in Fig. 1, the observed writing errors were partial response and phonologic and morphologic errors in both kana and kanji. In addition to

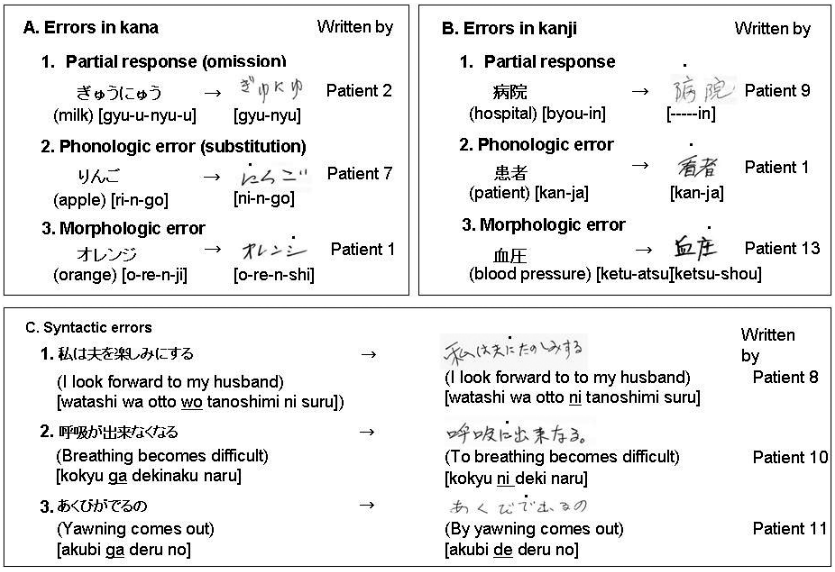

Fig. 1. Samples of writing errors observed in the study. Samples of writing errors in kana (A) and kanji (B) are shown in the upper half of the figure. For each type of error the correct text is typed on the left of the arrow and the samples written by the patients themselves are on the right with the wrong letters indicated by dots for all errors except omission. The corresponding English and the pronunciation for correct and incorrect text are shown below each word in the parentheses and blankets, respectively. Writing errors in kana, included partial responses, with omission of one or more kanas that constitute a kana word (A-1); phonologic errors, in which one or more kanas that constitute a kana word is substituted for other kanas (A-2); and morphologic errors, with omission or addition of a component of kana (A-3). Writing errors in kanji included partial responses, in which part of the kanji components were correct but the written kanji does not really exist (B-1); phonologic errors, with substitution of another kanji that has the same value as the correct component but a different meaning (B-2); and morphologic error, with substitution of another kanji that has visual resemblance to the correct component (B-3). Samples of syntactic errors are shown in the lower half of the figure (C). Mistakes with postpositions (prepositions) are indicated by dots in each sentence written by patients and the corresponding pronunciation is underlined. Mistakes included misuse of a postposition that modifies an indirect object instead of a postposition that modifies a direct object (C-1); misuse of a postposition that modifies an indirect object instead of a postposition that modifies a subject (C-2); and misuse of a postposition that modifies an adverb instead of a postposition that modifies a subject (C-3).

these errors, some patients showed misuse of kana letters mainly for postpositions, thereby suggesting syntactic errors. The most frequent type of writing errors was the partial response (omission) of kana letters observed in 11 patients, followed by phonologic errors (substitution) of kana in 8 patients, and syntactic errors in 6 patients (Table 2). Non-response and semantic errors were not observed and there were no apraxic or spatial features of writing errors in any patients.

### 3.3. *Characteristics of SPECT images*

The characteristics of SPECT images acquired from 14 patients exhibited feature of reduced uptake in the bilateral frontal lobes beyond the primary motor cortex (Table 3). Reduced uptake was also observed frequently in the temporal lobes and occasionally in the parietal lobe. Uptake in the occipital lobes was preserved in all patients. Reduced uptake was predominant on the left-side in 8 patients and symmetrical in 6 patients; none of the patients showed right-sided predominance of reduced uptake.

## 4. DISCUSSION

Cases of ALS presenting with progressive aphasia have been reported as a rare condition and most cases are preceded by aphasic syndrome followed by motor symptoms with predominant bulbar features, namely bulbar-onset ALS [4–7,10,17,22,29,38]. Some writing errors have been mentioned as a part of aphasia [5,6, 10,17,38], but less attention has been paid to writing problems. Our study is the first in which writing errors were investigated in a number of patients with bulbar-onset ALS, and we found a high prevalence of writing errors in these patients. This prevalence may not be generally applicable to patients with ALS due to the study design, but our observation suggests that writing errors are common feature in patients with bulbar-onset ALS. Writing errors were first encountered from 3 to 40 (mean 18.6 $\pm$ 13.3) months after the onset of bulbar symptoms, although these errors might have existed prior to the evaluation of writing. At the time when writing errors were encountered, co-development

Table 3
Summary of SPECT analysis

| Patient | Area of reduced uptake | | | | | | | | Dominant side of reduced uptake |
|---|---|---|---|---|---|---|---|---|---|
| | Right | | | | Left | | | | |
| | F | T | P | O | F | T | P | O | |
| 1 | + | + | | | + | + | + | | Left |
| 2 | + | + | | | + | + | | | Symmetric |
| 3 | + | + | | | + | + | + | | Left |
| 4 | + | + | | | + | + | + | | Left |
| 5 | + | + | + | | + | + | + | | Left |
| 6 | + | + | | | + | + | | | Symmetric |
| 7 | + | + | | | + | + | + | | Left |
| 8 | + | + | + | | + | + | + | | Left |
| 9 | + | + | | | + | + | | | Symmetric |
| 10 | + | | | | + | | | | Symmetric |
| 11 | + | + | + | | + | + | + | | Left |
| 12 | + | + | | | + | + | + | | Left |
| 13 | + | + | + | | + | + | + | | Symmetric |
| 14 | + | + | | | + | + | | | Symmetric |
| Total | 14 | 13 | 4 | 0 | 14 | 13 | 9 | 0 | |

Abbreviations. F = frontal; T = temporal; P = parietal; O = occipital.

of FTD-like behavioral changes were observed in 10 of 15 patients, but features of dementia were not present in the other 5 patients, one of whom subsequently developed behavioral changes suggesting FTD. Thus, development of dementia after the onset of ALS symptoms was frequent in this study, similarly to a report indicating the possible underestimation of developing dementia in bulbar-onset ALS [31], and writing errors might precede overt dementia. It was of note that two patients (patients 1 and 2) showed isolated writing errors without either aphasia or dementia, indicating features of "pure agraphia". These findings suggest that writing errors can develop independently from or precede clinical manifestation of dementia/aphasia in some patients. Interestingly, Luzzi et al. [25] documented a patient with slowly progressive isolated pure dysgraphia who initially presented with only omission of letters and then developed apraxia of speech after 7 years. The authors speculated that the disease was frontotemporal lobar degeneration without dementia [25]. Some cases with peripheral progressive agraphia have also recently been reported in the context of corticobasal degeneration [12–14], although they had features of apraxic agraphia, in contrast to our ALS patients. Fukui et al. [12] stressed that progressive agraphia can be a harbinger of degenerative dementia, and we believe that the different types of agraphia documented in our study may be an early sign for development of dementia/aphasia in ALS.

Writing errors in ALS, in Western countries, first documented by Ferguson and Boller in 1977 [9]. They reported 2 cases of bulbar-onset ALS, one of whom was neuropathologically confirmed to have ALS, presenting with syntactic writing errors in sentences and spelling errors of words [9]. Although their speech was almost abolished and detailed neuropsychological evaluations were not performed, the features of writing errors were described as "pure agraphia" [9]. However, subsequent descriptions of writing errors in ALS patients did not appear until the late 1990 s [4–6,10,22,29], with a major focus on aphasia. These reports documented progressive aphasia combined with MND/ALS, and provided descriptions of spelling errors or paragraphia [6, 10,29], and syntactic errors [4]. Similarly to our observations, recent Japanese case reports have described the omission of kana, paragraphia, and syntactic errors [17,19,20], and all the patients in these reports appear to have features of bulbar-onset ALS. Among these reports, Kanzaki et al. [20] reported a patient with bulbar-onset ALS, who presented omission of kana but lacked overt dementia, subsequently developed apparent dementia, although his speech was incomprehensible and evaluation of aphasia was difficult. Our study is the first to indicate that partial response (omission) of kana is the most frequent and common writing errors in ALS patients with bulbar-onset, with or without dementia/aphasia.

The SPECT images acquired in the study did not reveal a detailed topographical correlation with each type of writing error, but involvement of the left frontotemporal lobe was a consistent feature in SPECT images. This suggests that dysfunction of this region may be associated with the pathomechanism underlying the writing errors. Considering the anatomical relationship, involvement of the frontal regions located adjacent to the primary motor cortex corresponding to the orolingual area may be important. Historically, the writing center is considered to be located at the foot of the middle frontal gyrus, the so-called Exner area [3,8,37]. Recently, functional magnetic resonance imaging (fMRI) studies have also suggested that the left middle frontal region is crucial to writing, along with other regions such as the intraparietal sulcus, cingulate gyrus, supplementary motor cortex, and posterior inferior temporal cortex [21,27,34]. An interesting study of direct cortical stimulation under awake surgery conducted in patients with a brain tumor showed that writing-specific interference was localized in the posterior part of the dominant middle frontal gyrus and that stimulation of this region elicited writing errors such as letter omission and paragraphia [24]. With regard to syntactic errors, Bak et al. [4] reported that patients with MND presented with impaired syntactic comprehension and selective impairment of verb processing and showed selective pathological changes in Brodmann areas 44 (Broca's area) and 45. Several neuropsychological studies and recent neuroimaging studies have also suggested that Broca's area plays an important role in the processing of syntax [11,35,39]. Other than the frontal lobe, it has also been documented that the posterior inferior temporal cortex is important in spelling [30]. Taken together, these findings suggest that writing errors are closely associated with the language-related frontotemporal lobe.

We recognize several limitations of the study, including the nature of the non-systematic retrospective study, the evaluation of writing errors without well-organized writing tests, the lack of a comparable investigation of limb-onset ALS patients, and analyses of SPECT images only visually and without control data for patients without writing errors. However, a systematic study with various neuropsychological tests is not practical clinically, since at the time of the first visit to neurologists spoken language is already poor and writing is the only way to communicate with many of patients with bulbar-onset ALS. Conversely, it is often difficult to evaluate writing in limb-onset ALS patients due to progressive hand disabilities. Regarding to the SPECT images, most of our patients without writing errors have not undergone SPECT, and therefore we were unable to investigate differences in imaging features between patients with and without writing errors.

Within these limitations, we consider that our results are consistent with previously documented evidence and that our observations provide further information for clinical practice and clinicopathological studies. Regarding to word retrieval impairment, recent studies using fMRI and $^{11}$C-flumazenil positron emission tomography have shown that poor performance on confrontation naming correlates with localized dysfunction in the prefrontal region, including the middle or inferior frontal gyrus extending to Broca's area [1,2]. These results indicate that language dysfunction is present in ALS patients, in addition to executive dysfunction, and suggest that this condition occurs due to selective involvement of extra-motor regions, rather than diffuse brain dysfunction [1,2]. These studies were conducted exclusively for patients with limb-onset ALS or without severe bulbar involvement [1,2], but the results are somewhat supportive of our conclusions, and *visa versa*. Respiratory dysfunction has also been argued to influence cognitive functions, but limb-onset ALS patients with respiratory failure do not necessarily develop dementia or aphasia. This does not explain the enigma of the close relationship between dementia/aphasic syndrome and bulbar-onset ALS, and we believe that writing errors are attributable to the primary pathological involvement of selected brain regions, as seen in a report of MND/ALS with aphasia [4], and are not due to secondary brain damage caused by respiratory dysfunction.

In conclusion, our results suggest that writing errors are the common clinical feature of bulbar-onset ALS and are not merely consequences of dementia/aphasia. Such writing errors may develop independently from or precede clinical manifestation of dementia/aphasia, and may involve frontotemporal language-related areas beyond the primary motor cortex.

## Acknowledgements

M.K. is supported by a grant from the Core Research for Evolutional Science and Technology (CREST, 17022035) and a Grant-in-Aid for Scientific Research on Priority Areas – System Study on Higher-order Brain Functions from the Ministry of Education, Culture, Sports, Science and Technology (MEXT, 18020027).
